# Medical education during the COVID-19 pandemic: a reflection on the JHUSOM experience

**DOI:** 10.1186/s12909-024-05266-9

**Published:** 2024-03-25

**Authors:** Sydney A. Wade, Iman Ali, Aaron M. Milstone, Sarah L. Clever, Shaoming Xiao, Danielle Winner Koontz, Bhakti Hansoti

**Affiliations:** 1 Johns Hopkins University School of Medicine, Baltimore, MD USA; 2 Division of Infectious Diseases, Department of Pediatrics, Johns Hopkins University School of Medicine, Baltimore, MD USA; 3 Division of General Internal Medicine, Department of Medicine, Johns Hopkins University School of Medicine, Baltimore, MD USA; 4 Department of Emergency Medicine, Johns Hopkins University School of Medicine, Baltimore, MD USA; 5 Department of International Health, Bloomberg School of Public Health, Baltimore, MD USA

**Keywords:** Undergraduate medical education, COVID-19, Medical students, Medical curriculum, Behavioral risks

## Abstract

**Background:**

We sought to understand the relative risk of COVID-19 infection and identify risk factors for infection to identify targets for mitigation among medical students.

**Methods:**

An observational cohort study of Johns Hopkins School of Medicine students was conducted from June 2020 to July 2021. Blood samples were collected and tested at three visits to assess for antibodies against SARS-CoV-2. Additionally, a questionnaire was administered at each visit to collect demographic information and assess potential social and behavioral risk factors.

**Results:**

264 students enrolled in the study, and 38 participants completed all study requirements by study end. Roughly 6% of the first- and second-year classes had a reported positive COVID-19 test compared to 5% of third- and fourth-year students. By visit 3, 92% of medical students had detectable antibodies against COVID-19 compared to 4% during the study enrollment period. From study enrollment to visit 3, there was a 10-fold increase in the percentage of students reporting attending large social gatherings and dining in restaurants.

**Conclusions:**

Overall, few COVID-19 cases were found among medical students, even those on clinical rotations. As the study progressed, students reported engaging in higher-risk social behaviors in conjunction with increasing vaccination rates among students.

## Introduction

**Fig. 1:**
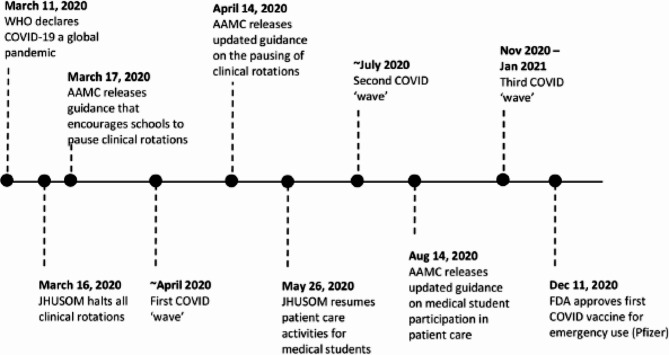
Timeline of curriculum changes during the early COVID-19 pandemic

The traditional organization and operations of undergraduate medical education were severely disrupted with the WHO’s declaration of COVID-19 as a pandemic in March 2020 [1] (Fig. 1). It is estimated that at least 165 MD- and DO-granting schools in the United States paused clinical rotations for third- and fourth-year students by the end of March [2]. This significant change in the delivery of medical education was largely driven by organizations such as the American Association of Medical Colleges (AAMC), which released guidance recommending the suspension of in-person clinical rotations starting March 17, 2020 [3]. While schools cited medical students’ safety as their top priority when making these curriculum changes, there were reports of the negative impacts of these changes on medical student well-being and clinical preparedness. A cross-sectional study of over 1,400 students across 40 medical schools found a 61% increase in anxiety and a 70% increase in depression among medical students during the COVID-19 pandemic [4]. Further, several students, particularly third- and fourth-years, reported concerns of not being able to meet graduation requirements, feeling inadequately prepared for away rotations or residency, or burning out upon entering the workforce [5, 6]. 

The decision to restructure medical school curricula (particularly in-person components) was initially based on the lack of quantification of COVID-19 infection risk and concerns about the lack of personal protective equipment (PPE) and COVID-19 testing for essential workers. With time, knowledge about SARS-CoV-2 transmission increased and access to PPE, COVID-19 testing, and later, vaccines expanded. Further, it become clear that the pandemic was no longer a short-term crisis but instead a new reality. These factors prompted schools to gradually reduce restrictions on in-person activities, including allowing students to return to their clinical rotations with safeguards in place to stymie SAR-CoV-2 transmission.

At the Johns Hopkins School of Medicine (JHUSOM), the pre-clerkship curriculum shifted onto virtual platforms on March 16th, 2020. Clinical rotations for third- and fourth-year students were also paused then but were re-launched in a condensed format on May 26, 2020. However, challenges persisted in fully integrating students into clinical care teams while following occupational safety guidelines related to COVID-19. At times, students and faculty noted a conflict between room occupancy limits, PPE availability, and large clinical teaching teams which traditionally have relied upon bedside learning.

JHUSOM, like many of its peer academic medical centers, made these unprecedented changes to their medical education system with the safety of their students, faculty, and patients in mind. However, it is unknown whether these efforts to reduce in-person contact were successful in reducing SARS-CoV-2 exposure among medical students, especially among third- and fourth-year students who were previously on clinical rotations. Therefore, this study was conducted to assess the risk of SARS-CoV-2 infection among medical students across various levels of training and identify potential risk factors associated with SARS-CoV-2 infection.

## Methods

### Study cohort

We conducted an observational cohort study of JHUSOM medical students from June 2020 to July 2021. All medical students enrolled at JHUSOM at the start of the study were eligible to participate, including students taking a research year or a leave of absence.

### Study setting

At JHUSOM, all lectures and small group discussion sessions were held over video conferencing for students in their pre-clerkship years starting in March 2020. Over time, the pre-clerkship curriculum gradually moved to a “flipped classroom” model, in which foundational concepts were taught through pre-recorded videos and bolstered with short in-person small group sessions. Clinical rotations for third- and fourth-year medical students were also initially suspended but were re-launched as condensed clerkships in May 2020. To augment the condensed clinical clerkships while reducing the educational burden on medical students, JHUSOM released new online elective courses and made all for-credit core credit courses pass-fail.

### Recruitment and consent

With permission from JHUSOM administration, a recruitment email was sent from the study email address to all JHUSOM medical students. The email introduced the study and included information about where students could find additional details about study. Students were also provided a link to the first REDCap form where they could complete the initial questionnaire and documentation of informed consent before receiving instructions about the blood draw. The study was approved by the JHUSOM Institutional Review Board (IRB00251625).

### Questionnaire administration

Participant data was captured in an electronic secure online database supported by Johns Hopkins University REDCap. The password-protected REDCap database hosted the consent form, the initial questionnaire, self-reporting of COVID-19 infection(s), and subsequent questionnaires.

The questionnaire collected the following data: demographic information (e.g., birth date, class year, race/ethnicity); living situation (e.g., number of housemates, including those working in a healthcare setting); clinical exposure (e.g., participation in patient care settings, current and past clinical rotations, and potential exposures on clinical rotations); influenza-like illness symptoms (e.g., history of COVID-19 and influenza testing, infection test results); and exposure to SARS-CoV-2 (e.g., number of exposures without a mask, frequency of high-risk behaviors, interaction with patients with known or unknown COVID-19 status). Additionally, any students who reported a potential COVID-19 diagnosis were encouraged to complete a follow-up survey, in which students were asked about dates and results of COVID-19 test results, current symptoms, and potential exposures. COVID-19 test results from participants were also queried from Epic.

Data was collected at three separate time points. At study enrollment, the questionnaires were collected from participants between June 2020 to October 2020. At visit 2, estimated to be roughly 4 months after study enrollment, the questionnaires were distributed from November 2020 to February 2021. Lastly, at visit 3, estimated to be roughly 8 months after study enrollment, the questionnaires were distributed from April 2021 to July 2021. These time points were chosen in anticipation of a peak in COVID-19 incidence in the fall of 2020.

### Sample collection and lab processing

All participants were asked to provide a blood sample at three various timepoints, roughly around the time of questionnaire completion. The first blood samples were collected from July 2020 to April 2021; the second from November 2020 to September 2021; and the third from February 2021 to August 2021. Participants were allowed to refuse repeat testing at any point. Samples were collected at Johns Hopkins affiliated laboratories and were tested for the presence of SARS-CoV-2 antibodies using enzyme-linked immunosorbent assays [7]. Any residual samples were stored at − 80 degrees after processing for potential repeat testing using next-generation tests.

All participants were able to access their test results through their Epic chart or by calling Johns Hopkins Hospital laboratory services. An FAQ document was provided to all participants to guide interpretation of test results.

### Data analysis

Descriptive statistics of the seroprevalence data and social and behavioral risk factors were calculated. To assess if there were significant changes in participant behavior from study enrollment to visit 3, p-values from the paired data were calculated using a McNemar test and corrected for multiple comparisons using the Bonferroni method. All data analyses were completed using R version 4.2 (Foundation for Statistical Computing, Vienna, Austria).

## Results

### Participant characteristics

During the study period, 264 enrolled in the study at the baseline, 167 remained enrolled at visit 2, and 76 remained enrolled at visit 3. Demographic information of the final cohort at study enrollment, visit 2, and visit 3 can be found in Table 1. At study enrollment, 113 (42.8%) of the 264 participants identified as male, 150 (56.8%) identified as female, and 1 (0.4%) identified as another gender. The four class years and students who were taking a research year or on leave were roughly equally represented with slightly more participation from the third- and fourth-year classes compared to the first- and second-year classes and the students on research year/leave of absence. Two (0.8%) of the study participants did not provide a class year. The median age for the cohort was 25.2 years with an interquartile range of 24.1 to 26.5 years.

**Table 1:** Study cohort at enrollment and visits 2 and 3

	No. of people at enrollment^a^ (%)*N* = 264	No. of people at visit 2^b^ (%)*N* = 167	No. of people at visit 3^c^ (%)*N* = 76
**Gender identity**			
Male	113 (42.8)	60 (35.9)	28 (36.8)
Female	150 (56.8)	107 (64.1)	48 (63.2)
Non-binary/other gender identity	1 (0.4)	0 (0.0)	0 (0.0)
**Class year**			
1st	46 (17.4)	26 (15.6)	18 (23.6)
2nd	43 (16.3)	24 (14.4)	10 (13.2)
3rd	68 (25.8)	47 (28.1)	26 (34.2)
4th	60 (22.7)	42 (25.1)	12 (15.8)
Research year/leave of absence	45 (17.0)	28 (16.8)	10 (13.2)
No response	2 (0.8)	0 (0.0)	0 (0.0)
**Race**			
American Indian or Alaska Native	0 (0.0)	0 (0.0)	0 (0.0)
Asian	90 (34.1)	57 (34.1)	25 (32.9)
Black or African American	9 (3.4)	3 (1.8)	2 (2.6)
Native Hawaiian or Other Pacific Islander	0 (0.0)	0 (0.0)	0 (0.0)
White	139 (52.7)	93 (55.7)	42 (55.3)
Multi-race	18 (6.8)	11 (6.6)	4 (5.3)
No response	8 (3.0)	3 (1.8)	3 (3.9)
**Ethnicity**			
Hispanic/Latino	23 (8.7)	14 (8.4)	7 (9.2)
Not Hispanic/Latino	230 (87.1)	149 (89.2)	65 (85.5)
No response	11 (4.2)	4 (2.4)	4 (5.3)
**Age (median, IQR)**	25.2 (24.1–26.5)	25.3 (24.0–26.5)	24.9 (23.9–26.6)

^a^Data collected from June 23, 2020 to Dec 05, 2020

^b^Data collected from Nov 12, 2020 to Aug 28, 2021

^c^Data collected from April 12, 2021 to Sept 11, 2021

At visit 2, 167 participants remained in the study. The proportion of female participants increased from 56.8% at enrollment to 64.1% (107/167) at visit 2. Other demographics, such as class year and median age, did not change significantly from enrollment to visit 2. Lastly, of the 76 participants who were still enrolled in the study at visit 3, 48 (63.2%) identified as female. The first- and third-year classes were more heavily represented amongst the remaining participants with 18 (23.6%) first years and 26 (34.2%) third years enrolled. The median age decreased slightly to 24.9 (interquartile range: 23.9 to 26.6).

### COVID-19 antibody seroconversion

At the time of study enrollment, 209 (93.3%) of the 224 study participants were seronegative, 6 (2.7%) were unvaccinated and IgG positive, and 9 (4.0%) were vaccinated and IgG positive (Table 2). At visit 2, 48 (40.3%) of the 119 remaining participants who provided a blood sample were seronegative, 11 (9.3%) were unvaccinated and IgG positive, and 60 (50.4%) were vaccinated and IgG positive. By visit 3, only 1 (2.6%) of the 38 remaining participants who provided a blood sample were seronegative. Only 2 (5.2%) were unvaccinated and IgG negative while the remaining 35 (92.2%) of the participants were vaccinated and IgG positive.

Of the students with COVID-19 PCR test results on file, 3/49 (6.1%) of the first- and second-year students tested positive for COVID-19 compared to 4/85 (4.7%) of third- and fourth-year students and 1/30 (3.3%) of students taking a research year or on leave (Table 3).

### Social and behavioral risk factors

At study enrollment, most of the participants lived with 1 or 2 other people while approximately 10% of participants lived alone (Table 4). Additionally, 151/262 (57.6%) of participants lived with at least 1 person who worked in healthcare (including other medical students).

**Table 2:** Seroprevalence and COVID testing at enrollment and visits 2 and 3

	1st blood sample^a^ (%)*N* = 224	2nd blood sample^b^ (%)*N* = 119	3rd blood sample^c^ (%)*N* = 38
**Seroprevalence**			
Unvaccinated + IgG negative	209 (93.3)	48 (40.3)	1 (2.6)
Unvaccinated + IgG positive	6 (2.7)	11 (9.3)	2 (5.2)
Vaccinated + IgG negative	0 (0.0)	0 (0.0)	0 (0.0)
Vaccinated + IgG positive	9 (4.0)	60 (50.4)	35 (92.2)

^a^Data collected from July 13, 2020 to April 16, 2021

^b^Data collected from Nov 18, 2020 to Sept 8, 2021

^c^Data collected from Feb 12, 2021 to Aug 30, 2021

**Table 3:** Positive COVID tests among students with PCR results by class year

	No. of 1st and 2nd years (*N* = 49)	No. of 3rd and 4th years (*N* = 85)	No. on research or on leave of absence (*N* = 30)
COVID-19 positivity (n, %)	3 (6.1)	4 (4.7)	1 (3.3)

**Table 4:** Social and behavioral risk factors of medical students by class year at study enrollment

	No. of 1st and 2nd year students (%)*N* = 89	No. of 3rd and 4th year students (%)*N* = 128	No. of students on a research year / leave of absence (%)*N* = 45	Total no. of students (%)*N* = 262^a^
**Home environment**				
**No. of people live with:**				
0	8 (9.0)	14 (10.9)	1 (2.2)	23 (8.8)
1–2	57 (64.0)	72 (56.3)	34 (75.6)	163 (62.2)
3–4	23 (25.9)	41 (32.0)	10 (22.2)	74 (28.2)
No response	1 (1.1)	1 (0.8)	0	2 (0.8)
**Lived with someone in in healthcare**:	41 (46.1)	80 (62.5)	30 (66.7)	151 (57.6)
**Non-clinical activities and exposures**				
**Participated in classroom activities in last 4 months**	21 (23.6)	23 (18.0)	6 (13.3)	50 (19.1)
**Travelled to state/country with community transmission in last 4 months**	53 (59.6)	69 (53.9)	20 (44.4)	142 (54.2)
**Clinical activities and exposures**				
**Exposed without a mask to COVID-19 positive**:				
Household contact	0	0	1	1
Non-work friend	1	0	1	2
Patient	0	1	1	2
Hospital staff member	0	0	1	1
None	83	125	41	249
**Had patient interaction in last 4 months**:	24 (27.0)	76 (59.4)	10 (22.2)	110 (42.0)
**Spent time with COVID-19 patients**:				
No	80 (89.9)	122 (95.3)	45 (100.0)	247 (94.3)
Yes	5 (5.6)	6 (4.7)	0 (0.0)	11 (4.2)
No response	4 (4.5)	0 (0.0)	0 (0.0)	4 (1.5)
**Percent of time spent with patients not known to have COVID-19**:				
0%	59 (66.3)	57 (44.5)	35 (77.8)	151 (57.6)
1–50%	21 (23.6)	18 (14.1)	2 (4.4)	41 (15.6)
51–100%	6 (6.7)	49 (38.3)	8 (17.8)	63 (24.0)
No response	3 (3.4)	4 (3.1)	0	7 (2.7)

^a^Excludes 2 students who did not indicate a class year

Amongst the 262 participants with a documented class year, there were 6 reported instances in which the participants had a maskless exposure to a person who was COVID-19 positive at study enrollment (June 2020 - October 2020). Of these 6 instances, 1 involved a household contact, 2 involved non-work friends, 2 involved patients, and 1 involved a hospital staff member. Additionally, at study enrollment, 110/262 (42.0%) participants reported having patient interactions within the last 4 months, of which 76 were third- and fourth-year students who would normally be in their clerkship year.

### Participant behavior change over time

**Fig. 2:**
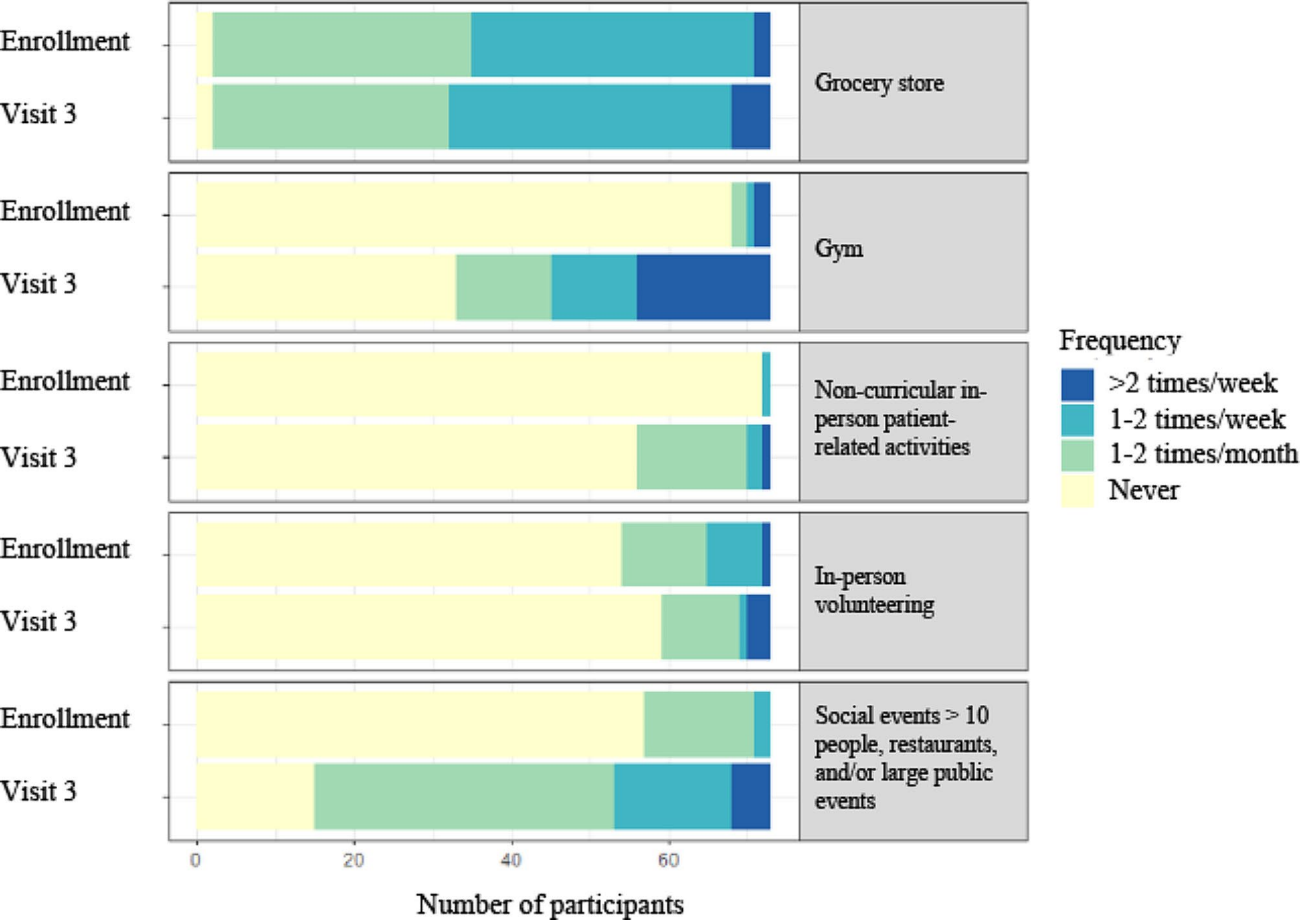
Change in participant behavior from study enrollment to visit 3

At visit 3, 76 participants completed the questionnaire, though 3 participants were excluded due to missing values in their questionnaires. Among the remaining 73 participants, significantly more participants reported attending the gym at visit 3 than at study enrollment (Fig. 2; Table 5). Additionally, significantly more participants reported attending social events with > 10 people, dining in restaurants, and/or attending a large public event. There was no statistically significant change in the number of participants who reported going to the grocery store, participating in non-curricular patient-related activities, or volunteering in person between study enrollment and visit 3.

## Discussion

In this longitudinal study assessing COVID-19 acquisition and exposures among medical students, we found low COVID-19 infection rates among our study participants, including the third- and fourth-year students working in clinical settings. Additionally, we found that many of the COVID-19 exposures occurred outside of clinical settings. Students at JHUSOM were also highly vaccinated (> 90%) likely accounting for the overall lower infection rates by visit 3. Furthermore, stringent university policies restricted clinical exposures for third- and fourth-year medical students, and many first- and second-year students opted to study and reside at homes outside of Baltimore, resulting in fewer on-campus interactions. Overall, we found that SARS-CoV-2 infection among medical students with direct clinical exposure was lower than what may have been initially anticipated by schools.

Out of the 264 students enrolled, there were only six reports of high-risk COVID-19 exposures at enrollment, of which half of these exposures occurred outside of clinical settings (e.g., with a household contact or non-school/work-related friend). This is consistent with what was found in a study of Jordanian medical students, which found that at least 50% of the students on clinical clerkships with positive tests also thought they were exposed outside of the hospital [8]. Taken together, these findings suggest that community exposures likely played a larger role in SARS-CoV-2 infection among medical students than clinical exposures. This hypothesis is supported by prior research conducted by Jacob et al. which found that community exposures, not workplace exposures, conferred the greatest risk for COVID-19 infection among healthcare workers in the US [9]. Further, a Danish study found that COVID-19 seroprevalence among medical students was more heavily associated with students’ social behaviors than with exposure to COVID-19-positive patients in clinical settings [10]. 

Our study also demonstrated that students’ social behaviors greatly changed over time. At study enrollment, fewer than 3% of students reported attending a large social gathering or dining in a restaurant more than 1–2 times per month. This number increased to almost 30% by visit 3.

**Table 5:** Change in participant behavior from study enrollment to visit 3

	Enrollment^a^ (%)*N* = 73		Visit 3^b^ (%)*N* = 73		p-value
Frequency (%)	Less frequent than 1–2 times/month	More frequent than 1–2 times/month	Less frequent than 1–2 times/month	More frequent than 1–2 times/month	
Grocery store	35 (47.9)	38 (52.1)	32 (43.8)	41 (56.2)	1.0
Gym	70 (95.9)	3 (4.1)	57 (61.6)	28 (38.4)	**< 0.001**
Non-curricular in-person patient related-activities	72 (98.6)	1 (1.4)	70 (95.9)	3 (4.1)	1.0
In-person volunteering	65 (89.0)	8 (11.0)	69 (94.5)	4 (5.5)	1.0
Social events > 10 people, dined in restaurant, large public event	71 (97.3)	2 (2.7)	53 (72.6)	20 (27.4)	**< 0.001**

^a^Data collected from June 2020 to October 2020

^b^Data collected from April 2021 to July 2021

This behavior change is notable because throughout the first year of the pandemic, large social gatherings and indoor dining were largely discouraged due to research that demonstrated these locations contributed to super-spreader events [11]. Therefore, the 10-fold increase in the percentage of students who frequented large social gatherings or restaurants suggests students’ perception of COVID-19 risk changed over time in our study, and that students were more likely to engage in social gatherings, even prior to vaccination being routinely available.

This change in students’ COVID-19 risk perception could have been driven by several factors. Of note, the percentage of students who were vaccinated against SARS-CoV-2 increased from 4% at enrollment to over 90% by visit 3 as access to COVID-19 vaccines expanded. Further, prior studies have shown that medical students believed that vaccination would help stymie SARS-CoV-2 transmission [12]. Therefore, it is likely that medical students at visit 3 felt more comfortable engaging in higher-risk behaviors than they would have at study enrollment because of the perceived protection they received from the COVID-19 vaccine or from prior infection as seen among healthcare workers [13]. Additionally, students may have reached “pandemic fatigue”, a phenomenon in which people become less adherent to public health interventions due to the perceived high burden on quality of life over time [14]. This pandemic fatigue could have contributed to students being less cautious about reducing their exposures to SARS-CoV-2.

This study suffered from high attrition of participants as the study progressed and possible enrollment bias. Since there weren’t any on-campus COVID-19 testing requirements for medical students, initially, students viewed this study as an opportunity to know their serostatus. As the study progressed and routine asymptomatic testing became more available, student participation decreased. Only 38/264 of the participants completed all three questionnaires and blood samples by visit 3. This high attrition rate could have introduced selection bias, especially if participants who tested positive for COVID-19 or engaged in higher-risk behavior were less likely to remain in the study. Further, we predict that students who continued to be tested for COVID-19 in our study may have been more likely to be unsure of their COVID-19 status compared to those who opted to leave the study. Our study may have also missed asymptomatic students who never sought out COVID-19 testing, students who received point-of-care testing, and students who got tested at non-Hopkins locations. Pandemic fatigue may have also contributed to the attrition rate, such that students who wanted to disengage from matters related to the pandemic may have been less likely to continue participating in our study.

Despite these limitations, our study’s observations raise the question of whether pausing and later condensing clinical rotations was necessary for reducing SARS-CoV-2 transmission among medical students. Outside of clinical and educational settings, students (like the general population) were responsible for their own decisions regarding their COVID-19 risk behavior, relying on their understanding and knowledge about SARS-CoV-2 transmission. However, schools took away students’ decision-making abilities in educational and clinical settings when they canceled in-person pre-clerkship and clerkship training, even though a study showed that two-thirds of medical students would have opted into clinical rotations [15]. School administrators made these decisions with the health of students, faculty, and patients in mind; there was concern that students may engage in high-risk behaviors outside of clinical and educational settings, which could pose a risk to contacts they interact with in clinical settings. However, these efforts to reduce in-person interactions did not come without consequences. With the onset of the COVID-19 pandemic, medical students who were already navigating non-academic stressors associated with the pandemic reported feeling additional strain associated with the dramatic changes to the medical curriculum [16]. With these changes, students, particularly those in their last years of training, were found to have higher rates of burnout and cynicism just prior to entering residency [17]. This could have adverse effects on their career trajectory, including increasing their desires to leave the medical field.This will likely not be the last pandemic that may significantly alter medical school operations. In future pandemics, we challenge medical school educators to weigh the potential infection-reducing benefits arising from significant changes in medical curriculum. We explicitly draw attention to the negative consequences associated with stopping or altering clinical rotations—namely reductions in student confidence and performance and increases in mental health concerns/symptoms.

## Conclusions

During the COVID-19 pandemic, the undergraduate medical curriculum at Johns Hopkins School of Medicine was drastically modified to reduce viral transmission within the community. In this study, we found few COVID-19 cases among medical students, including those that were on modified clinical rotations. Most of the potential COVID-19 exposures occurred in non-clinical settings, suggesting these settings were the main drivers to transmission, not clinical environments.

## Data Availability

The datasets used and/or analyzed during the current study are available from the corresponding author on reasonable request.

## Abbreviations

AAMC: American Association of Medical College
JHUSOM: Johns Hopkins University School of Medicine
PPE: Personal protective equipment
